# Cisternostomy as a Surgical Treatment for Traumatic Brain Injury-Related Prolonged and Delayed Intracranial Pressure Elevation: A Case Report

**DOI:** 10.7759/cureus.37508

**Published:** 2023-04-12

**Authors:** Akram M Eraky, Randall Treffy, Hirad S Hedayat

**Affiliations:** 1 Department of Neurosurgery, Medical College of Wisconsin, Milwaukee, USA

**Keywords:** lilliquist's membrane, basal cistern, lamina terminalis, advances in traumatic brain injury management, tbi, cisternostomy

## Abstract

Traumatic brain injury (TBI) can be classified into primary, due to the effect of the initial trauma, or secondary, due to increased intracranial pressure (ICP). Increased ICP may cause brain herniation and also decreases cerebral blood perfusion leading to ischemia. Recently, a few studies showed that cisternostomy with decompressive craniectomy (DC) has better outcomes than DC alone in patients with TBI. This can be explained by the recent advances indicating that cisternal cerebrospinal fluid (CSF) communicates with cerebral interstitial fluid (IF) through Virchow-Robin spaces. Theoretically, opening cisterns to atmospheric pressure may induce IF drainage and subsequently decrease ICP. A 55-year-old man presented to the emergency department with subdural hematomas, hemorrhagic contusions, and subarachnoid hemorrhage after falling off a moving truck. ICP elevation was refractory despite increased sedation, initiation of paralysis with Cisatracurium, esophageal cooling, multiple doses of 23.4 % saline and mannitol, and DC. Lumbar drain (LD) placement was performed with beneficial results. Unfortunately, the LD stopped functioning multiple times and each time this occurred, he developed increased ventricular size with elevated ICP. The patient underwent cisternostomy and lamina terminalis fenestration. No further increased ICPs were observed after cisternostomy at a one-month follow-up. Cisternostomy is a potential surgical treatment for patients with TBI-related prolonged ICP elevation.

## Introduction

Traumatic brain injury (TBI) includes multiple pathologies, such as epidural hemorrhage, subdural hemorrhage, subarachnoid hemorrhage, cerebral contusions, skull fractures, cerebral edema, and diffuse axonal injury. The initial trauma is the primary insult with the resultant secondary injury and intracranial pressure (ICP) elevation [1]. Increased ICP may cause brain herniation, decrease cerebral blood perfusion, and induce ischemia [2,3]. Decreasing ICP to prevent secondary injuries is one of the mainstays of management in patients with TBI. Decompressive craniectomy (DC), hyperventilation, osmotic diuretics, antihypertensive drugs, sedatives, paralytics, and cerebrospinal fluid (CSF) drainage are different modalities to decrease ICP in patients with TBI [1,2,4].

A recent potential management for patients with TBI is cisternostomy with DC. Cisternostomy with DC has been shown to have better outcomes than DC alone in patients with TBI [5-7]. Recent advances indicate that cisternal CSF communicates with cerebral interstitial fluid (IF) through Virchow-Robin spaces. Theoretically, opening cisterns to atmospheric pressure may induce IF drainage and subsequently decrease ICP [5,7,8].

## Case presentation

**Figure 1:**
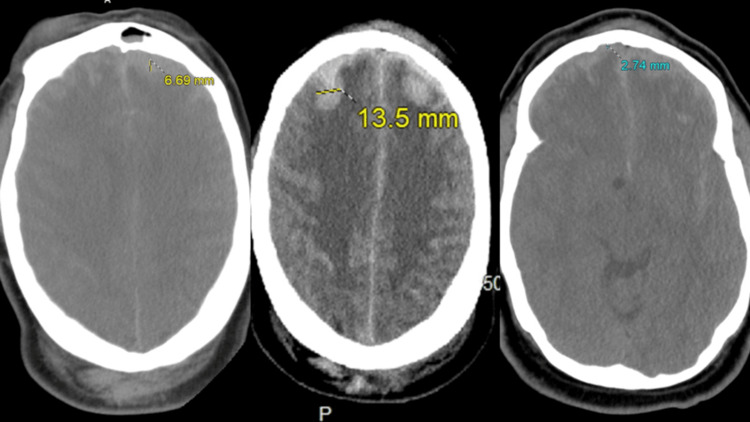
Axial head CT Head CT showing thin bifrontal subdural hematoma and subarachnoid hemorrhage with focal areas of hyperdensities along the inferior frontal lobes concerning for hemorrhagic contusions. CT, computed tomography.

 A 55-year-old man presented to the emergency department (ED) with significant facial and head trauma after falling off a moving truck. He had severe headaches and confusion; otherwise, the neurological exam was grossly intact with a Glasgow coma scale (GCS) of 14. His past medical history was unremarkable except for hypertension and alcoholism. Head computed tomography (CT) showed multiple facial fractures, thin bifrontal subdural hematomas (SDHs), and subarachnoid hemorrhage with focal hyperdensities along the inferior frontal lobes concerning hemorrhagic contusions (Figure 1).

**Figure 2:**
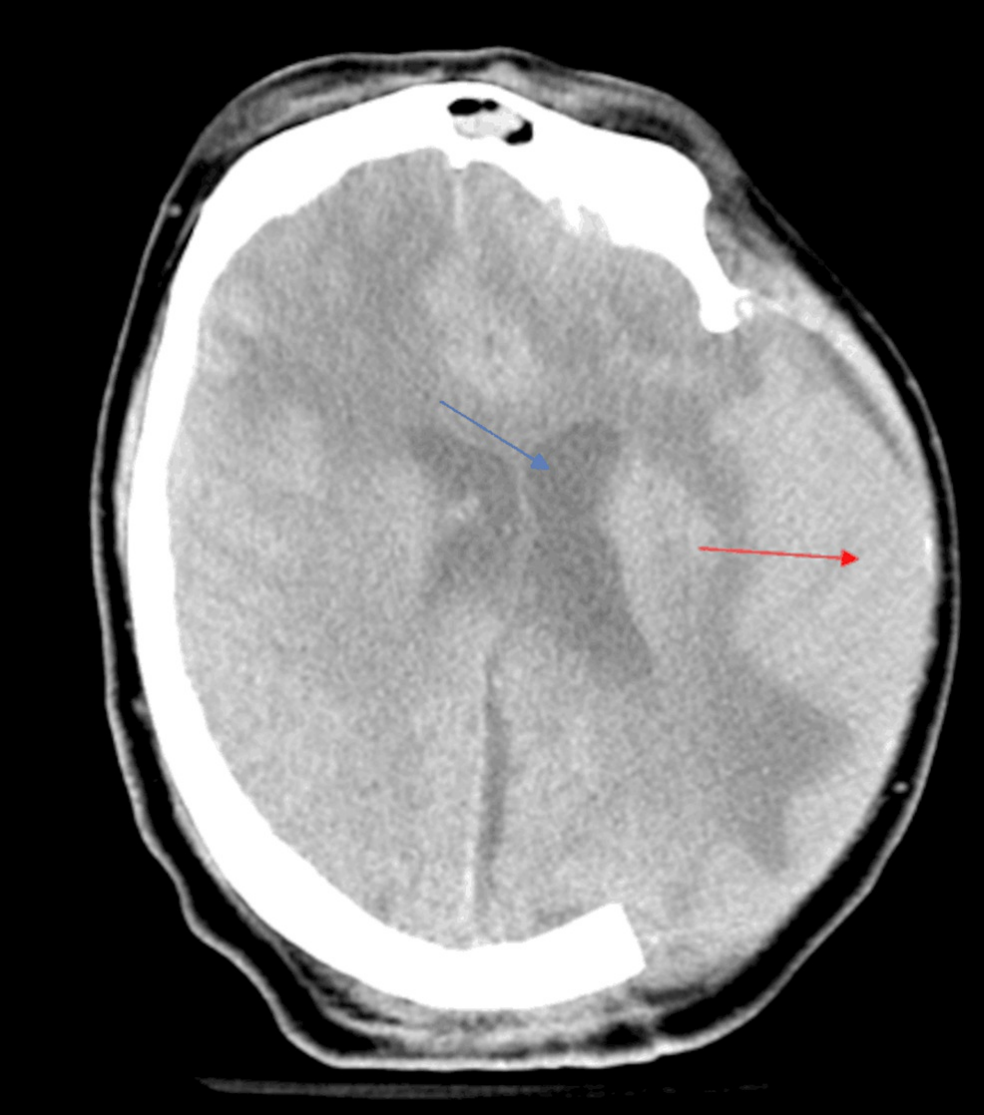
Axial head CT The view shows increased ventricular size (blue arrow) and parenchymal protrusion through the craniectomy defect (red arrow) with diffuse parenchymal edema in bilateral frontal lobes when the lumbar drain was not functioning. CT, computed tomography.

One day later, he experienced acute respiratory decompensation resulting in intubation, and a progression of hemorrhage was noticed on the scheduled repeat CT head. External ventricular drain (EVD) was placed with an initial ICP of 50 mm Hg. Increased sedation with intermittent mannitol and hypertonic saline was administered to meet cerebral perfusion pressure and ICP goals. ICP elevation was refractory despite increased sedation, initiation of paralysis with Cisatracurium, esophageal cooling, and multiple doses of 23.4% saline and mannitol. Next, left DC was performed after maximizing medical management. ICPs remained elevated after DC and thus lumbar drain (LD) placement was performed with beneficial results. Unfortunately, the LD stopped functioning multiple times, and each time this occurred, he developed increased ventricular size with elevated ICP (Figure 2). His CT also demonstrated increasing parenchymal edema each time the LD malfunctioned in conjunction with elevated ICP. This process was recurring during the second and third weeks of his hospitalization.

Given that the patient was developing increasing ventricular size with refractory increased ICP and the family declined ventriculoperitoneal shunt as the next step in management, the risks versus benefits of a craniotomy for cisternostomy and fenestration of lamina terminalis were discussed with the patient's family in order to provide prolonged ICP and edema control to allow cranioplasty in a shorter time frame given his ongoing herniation through his craniectomy defect.

**Figure 3:**
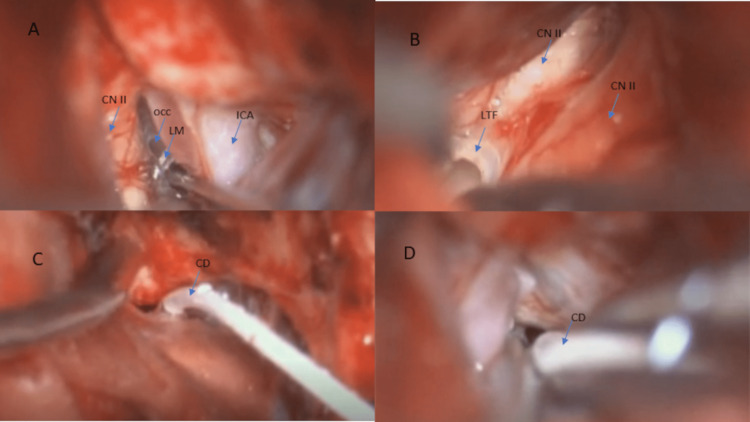
Cisternostomy (A) The view shows internal carotid artery (ICA), optic nerve (CN II), opticocarotid cistern (OCC), and Liliequist's membrane (LM) between ICA and CN II. (B) The view shows the ipsilateral and contralateral optic nerves (CN II), and lamina terminalis fenestration (LTF). (C) and (D) The two views show a catheter drain (CD) placed within the prepontine cistern.

**Figure 4:**
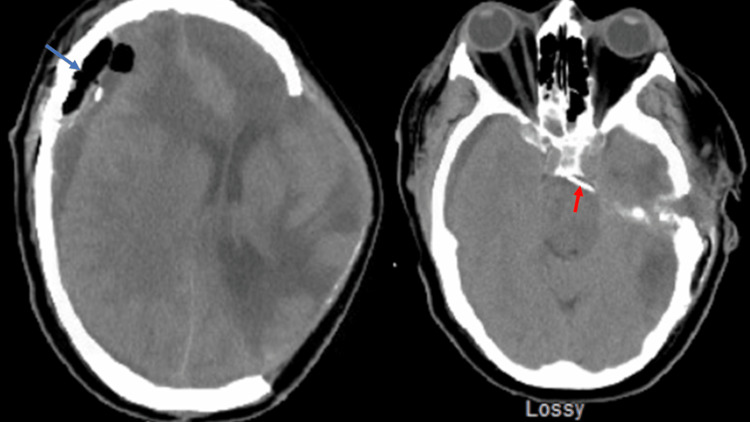
Postoperative axial CT. The view shows extra-axial fluid thought to be CSF (blue arrow) and the inserted catheter in the prepontine cistern (red arrow). CT, computed tomography.

A right frontotemporal craniotomy was created and the arachnoid was sharply dissected over the optic nerve as well as the internal carotid artery and the opticocarotid cistern. As expected, the arachnoid was quite adherent and thickened given that the injury had occurred a month prior. The membrane of Lilliquist was incised and the basilar artery was visualized after the arachnoid was widely opened. Finally, the interoptic cistern was sharply opened as were the contralateral opticocarotid cistern and lamina terminalis. Next, a catheter was placed within the prepontine cistern under direct visualization with the microscope. The catheter was then tunneled out of the skin rostrally and connected to a ventricular drainage system (Figure 3). Postoperatively, there were no complications. Head CT showed extra-axial fluid thought to be CSF (Figure 4).

**Figure 5:**
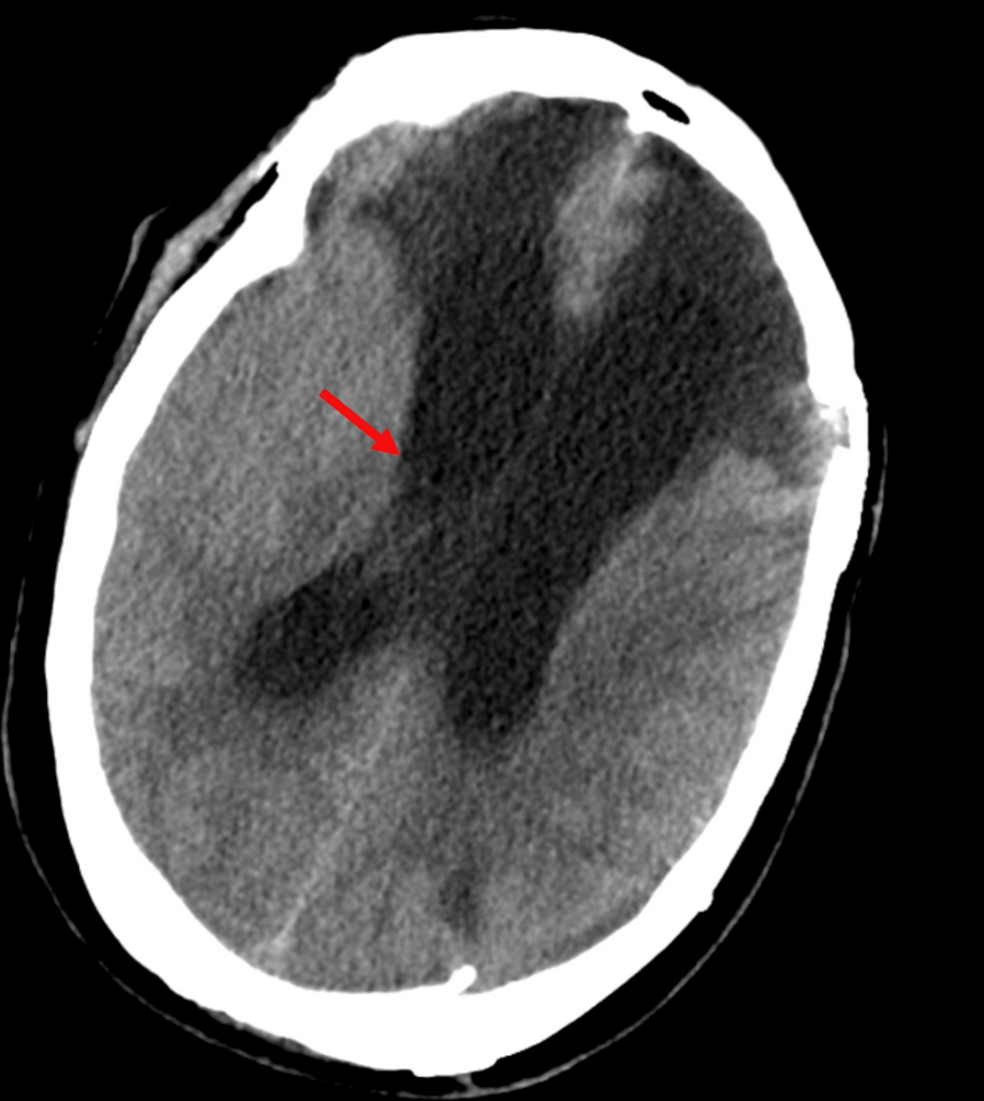
Axial CT head. The view shows post-traumatic hydrocephalus (red arrow). CT, computed tomography.

Five days later, the patient underwent left autologous cranioplasty. Postoperatively, the patient developed right-to-left shift with dilated pupils due to right acute on chronic SDH. Subsequently, he underwent a right redo craniotomy for hematoma and subdural drain. Twenty days later, EVD was placed to monitor ICPs with an opening pressure of 15 cm. The patient had a GCS of 14 with no further spikes of increased ICPs. At the one-month scheduled follow-up, the patient developed post-traumatic hydrocephalus, and a ventriculoperitoneal shunt was performed without issues (Figure 5).

## Discussion

One of the most important parameters to monitor in the management of TBI is ICP because it affects brain perfusion and can cause secondary brain injuries, such as ischemia and brain herniation [2]. DC is a surgery in which a bone flap is removed to allow for more space to decrease ICP [3]. In a recent clinical trial, Chandra et al. found that patients treated with DC and cisternostomy have lower complication rates and better outcomes than DC alone [7]. In another retrospective study, Giammattei et al. found that patients treated with DC and cisternostomy had better clinical outcomes [6]. The same group described cisternostomy’s cadaveric dissection and surgical technique in a separate paper [5]. A previous case report by our group demonstrated the effect of cisternostomy in decreasing ICP and its potential role as a surgical treatment for pseudotumor cerebri [8].

**Figure 6:**
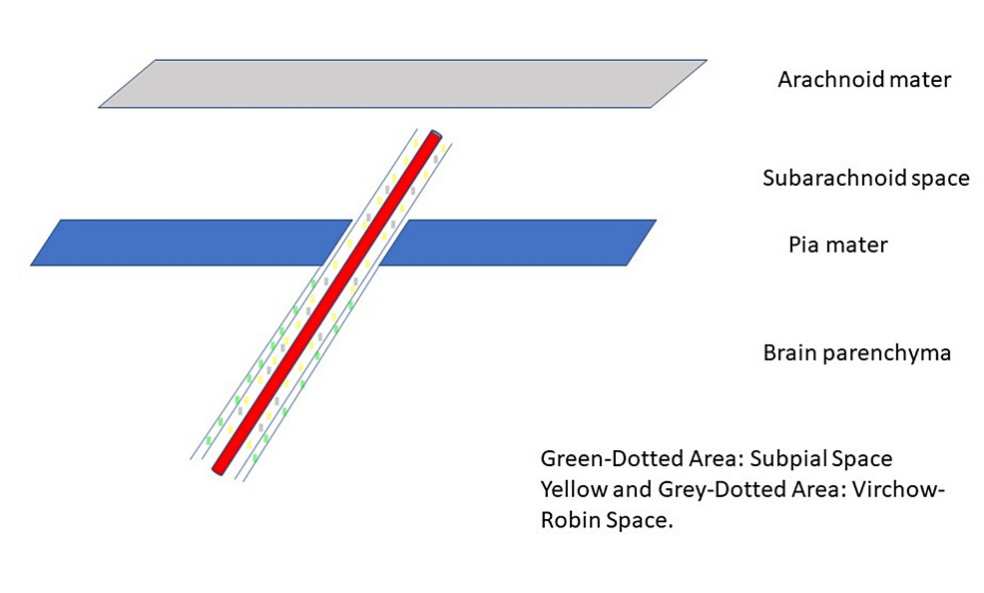
Virchow-Robin Space An illustration showing a cortical artery surrounded by the Virchow-Robin space. The Virchow-Robin space is a perivascular space passing through the subarachnoid and subpial spaces to the brain parenchyma. Virchow-Robin spaces surround arteries, arterioles, veins, and venules [11,12]. Image Credit: Akram M. Eraky.

Recent advances indicate that ventricular CSF does not communicate with parenchymal IF [5,6,8]. As a result, CSF diversion from the ventricular system does not have a strong effect in increasing IF drainage and subsequently decreasing edema and ICP. In contrast, cisternal CSF communicates directly with IF via the glymphatic system through the Virchow-Robin spaces, which are perivascular spaces passing through the subarachnoid and subpial spaces to the brain parenchyma (Figure 6) [6,9-12]. Based on this, opening basal cisterns to atmospheric pressure and inducing CSF drainage from the cisterns through a drain will stimulate CSF diversion from IF to the cisterns. Theoretically, this may decrease ICP and brain edema. In the case of TBI, this may prevent secondary injuries induced by increased ICP, such as brain herniation and ischemia. Herein, we present a case of TBI with increased ICP and parenchymal fullness with herniation through the craniectomy defect. At the one-month follow-up after cisternostomy, there were no further ICP issues.

## Conclusions

Cisternostomy is a potential surgical treatment for patients with TBI-related prolonged ICP elevation. Here, we present a case of TBI with persistent increased ICP for longer than one month after the initial injury. No further ICP elevations were observed after cisternostomy. Larger randomized multicenter trials are encouraged to assess the effectiveness of cisternostomy in cases of TBI-related prolonged ICP elevation.
